# Low-Cost, Low-Power Edge Computing System for Structural Health Monitoring in an IoT Framework

**DOI:** 10.3390/s24155078

**Published:** 2024-08-05

**Authors:** Eduardo Hidalgo-Fort, Pedro Blanco-Carmona, Fernando Muñoz-Chavero, Antonio Torralba, Rafael Castro-Triguero

**Affiliations:** 1 Department of Electronic Engineering, University of Seville, 41092 Seville, Spain; ehidalgo@us.es (E.H.-F.); pblanco@us.es (P.B.-C.); torralba@us.es (A.T.); 2 Mechanics of Continuous Media and Theory of Structures, University of Córdoba, 14071 Córdoba, Spain; me1catrr@uco.es

**Keywords:** edge computing, SHM, IoT, wireless sensor network

## Abstract

A complete low-power, low-cost and wireless solution for bridge structural health monitoring is presented. This work includes monitoring nodes with modular hardware design and low power consumption based on a control and resource management board called CoreBoard, and a specific board for sensorization called SensorBoard is presented. The firmware is presented as a design of FreeRTOS parallelised tasks that carry out the management of the hardware resources and implement the Random Decrement Technique to minimize the amount of data to be transmitted over the NB-IoT network in a secure way. The presented solution is validated through the characterization of its energy consumption, which guarantees an autonomy higher than 10 years with a daily 8 min monitoring periodicity, and two deployments in a pilot laboratory structure and the Eduardo Torroja bridge in Posadas (Córdoba, Spain). The results are compared with two different calibrated commercial systems, obtaining an error lower than 1.72% in modal analysis frequencies. The architecture and the results obtained place the presented design as a new solution in the state of the art and, thanks to its autonomy, low cost and the graphical device management interface presented, allow its deployment and integration in the current IoT paradigm.

## 1. Introduction

As it is well known, historically, society has been and is dependent on civil structures and, therefore, maximizing their useful life becomes a strategic objective for its prosperity. Structural design codes have proven to be successful over the years. However, once built, structures can weaken because they are sometimes subjected to heavy loads and severe working conditions or subjected to major seismic events. Therefore, early detection and assessment of this damage is necessary to ensure that structures continue to meet safety standards, which is the main objective of structural health monitoring (SHM). SHM technologies and systems have been appearing for the last two centuries, and during this time, they have undergone an important evolution that has improved their results and brought them closer to the achievement of the desired paradigm: accurate, real-time, low-cost and unattended monitoring. Thus, the first systems that applied Non-Destructive Evaluation (NDE) [1] consisted of monitoring deteriorations [2] in infrastructures after the detection of important seismic events in search of possible damage, generally under the OMA (Operational Modal Analysis) technique [3,4]. The sequence of seismic events, especially those that occurred in Northridge in 1994 and Kobe in 1955 [5], highlighted the vulnerability of civil structures to this type of event, as well as the high cost of both structural damage assessment and measuring devices [6], the difficulty and danger of accessing certain monitoring areas, and the time required to obtain bureaucratic permits to access these structures, especially those belonging to historical heritage. Historically, due to the complexity and size of civil infrastructures as well as the high cost of traditional high-precision devices, only a few wired sensors were deployed. However, reliable structural monitoring requires the deployment of a large number of devices along the structures [5], which is beginning to be solved with the emergence of wireless sensor networks (WSNs) [6,7,8,9], the decrease in the cost of electronic devices, the emergence of Micro Electronic Mechanical Systems (MEMSs) and their application to SHM [1,2,3,4,5,6,7,8,9,10,11,12,13,14,15], and the subsequent emergence of the Internet of Things (IoT) paradigm that enables wireless, low-cost and unattended monitoring. In addition, there are other monitoring options such as visual [16,17] or fibre optic-based [18,19,20], which usually involve high costs compared to the proposed solution and do not allow wide deployment or methods based on electro-mechanical impedance monitoring [21,22], which usually require sampling systems for analogue signals of several hundred kHz, involving the use of more expensive microcontrollers and peripherals than the system presented here. Therefore, they are outside the scope of this study. With this established framework, the interest of the research community appears to be in SHM solutions within the low-cost IoT paradigm, such as [14,23,24,25], which offer wireless monitoring of civil structures through independent location monitoring, i.e., without a temporal correlation between the data acquired by the different sensor nodes deployed in the structure, which allows frequency peak detection analysis to be carried out [26], but they do not satisfy the necessary requirements for the application of OMA and the detection of structural vibration modes, which is an important functional limitation. In this sense, since the emergence of WSNs, solutions for wireless time synchronization of measurement nodes have been proposed through technologies such as ZigBee, or, more recently, GPS signals in wireless and low-power devices [27]. On the other hand, it is important to note that the application of OMA involves the monitoring of large acceleration time series, which implies the wireless transmission of large amounts of data (in the order of 1 megabit per time series). This results in a significant limitation of the autonomy of the measurement devices, as wireless transmission usually has a very high impact on the total power consumption of these devices [27]. However, the emergence of very-low-cost microprocessors with high computational capacity allows the application of edge computing techniques that, thanks to local processing, allow for a reduction in the amount of information through the wireless network without compromising the quality of the monitored signals and without affecting the accuracy of the overall system, which is a technological challenge to overcome. This work is presented as an evolution in the authors’ line of research in the field of structural health monitoring. At this point, it is important to highlight the conception of this publication as an important evolution of the solution presented in [27], where a complete and synchronous (using GPS signal) solution for structural health monitoring is presented. For this reason, some ideas from the initial solution are included in this publication to make the presented solution self-contained. Building upon this foundation, the present work introduces several original and innovative implementations:

- Firstly, a new firmware architecture has been developed, enabling integration into the standardized IoT layer stack and its application to the SHM field.
- Additionally, another contribution is the implementation of the Decrement Technique (RDT) and its application to SHM, effectively reducing the amount of information to be transmitted wirelessly. As a result, the autonomy of the deployed devices in the field is maximized.
- From the perspective of the application server, an IoT big data architecture has been implemented. This architecture facilitates the direct application of structural identification algorithms and high-computational-capacity artificial intelligence techniques. These advancements enable more robust and efficient analysis of structural health data.

Therefore, this work presents a low-power SHM system that allows accurate, low-cost and unattended monitoring over long periods of time thanks to the application of the RDT. Furthermore, the system incorporates time synchronization through GPS signals, enabling the application of Operational Modal Analysis (OMA) techniques. It also leverages NB-IoT wireless connectivity, whose operators guarantee its maintenance and high levels of coverage in both urban and rural areas. This system is presented as a complete solution in terms of the functional layers defined for the IoT standard, which guarantees its integration into existing information systems and allows potential extensions with value-added services through the designed communication Application Program Interface (API). Finally, the solution is validated through a comparison of its results with those provided by two commercially accurate systems, deployed in a pilot structure at the Engineering School of the University of Seville and the Eduardo Torroja bridge in Posadas (Spain) [28]. The results obtained show a TRL7 (Technology Readiness Level) which positions the presented work as a solution susceptible to be certified as a next step to be part of the market catalogue.

## 2. Material and Methods

**Figure 1:**
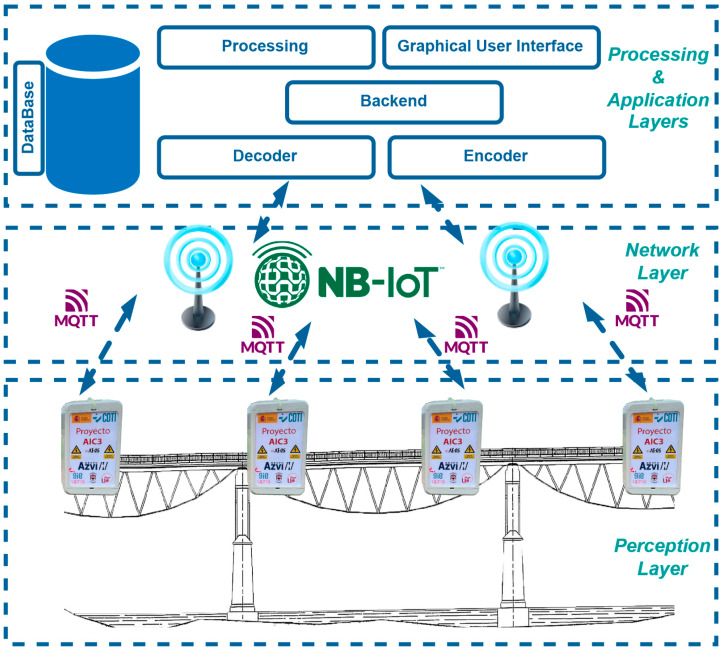
System overview.

As illustrated in Figure 1, this work presents a comprehensive SHM solution in terms of the stack of layers in IoT devices: the perception layer, network layer, and application and processing layers. The latter is implemented in the cloud application server that exploits the information received and enables the management of the monitored infrastructures.

- The perception layer consists of a series of low-power wireless monitoring nodes with local processing capabilities that enables edge computing capabilities, which are responsible for collecting the physical quantities related to the behaviour of the structure. This solution proposes the use of Random Decrement Time (RDT) to minimize the amount of data to be transmitted wirelessly and therefore reduce the amount of energy consumed. In addition to local processing, these samples will be processed in the application server to determine the modal characteristics of the structure.
- Network layer: this is the infrastructure in charge of establishing a bidirectional communication channel between the nodes of the perception layer and the application layer using NB-IoT technology.
- Processing layer: This is the layer composed of the infrastructure that houses the application layer as well as the necessary elements, both hardware and system, for its correct operation. It is responsible for integrating the non-relational database services, the processing backend, the data processing engine for both static and machine learning algorithms, and the network services.
- Application layer: This is the set of applications deployed in the processing server that add value to the information collected by the perception layer to offer it to the user, who is responsible for managing the monitored infrastructure. In the presented solution, it is made up of both the decoder/encoder that is responsible for encrypting/decryption the information in the uplink/downlink direction and the user interface and the processing algorithms housed in the processing layer.

Each of the layers discussed above will be described in detail in Section 2.1.

### 2.1. System Design

#### 2.1.1. Perception Layer

As its name indicates, the perception layer oversees acquiring information from the monitored structure through its sensors. It is, therefore, the one that has physical contact with the structure under analysis. On the other hand, its role involves preprocessing the acquired information and making it available to the next level layer, the network layer. This allows for the transmission of the processed data to the application layer. The proposed perception layer is built upon the development of both a hardware platform and the corresponding firmware that governs its operation.

**Figure 2:**
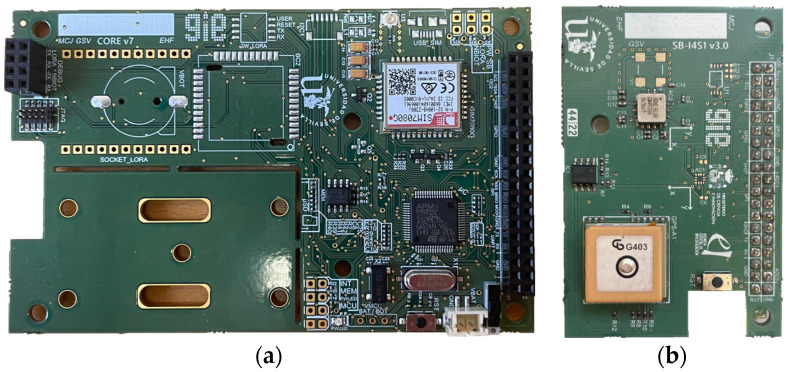
Hardware design: (**a**) CoreBoard; (**b**) SensorBoard.

Hardware Design: Given the heterogeneous nature of monitoring structural health in bridges in terms of the magnitudes to be acquired, a modular design is proposed with the capacity to adapt to different needs in terms of sensing and communication. For this reason, we have chosen a development that integrates, on the one hand, a board in charge of controlling the node and storing, processing and sending information, called CoreBoard, and another board responsible of providing the different acquisition and communication interfaces with the sensors, called SensorBoard. As mentioned above, one of the fundamental requirements of IoT devices is the minimization of their power consumption, which is impacted by both hardware and firmware design. From the hardware point of view, for the CoreBoard, shown in Figure 2a, the STM32L152RE microprocessor from STMicroelectronics with ARM Cortex-M3 architecture has been selected due its low power consumption and the wide catalogue of interfaces it offers. These characteristics guarantee communication with the desired sensors in each specific application. Additionally, as stated in [29], the analysis of modal parameters of bridges based on the frequency response requires the processing of time series to be 1000 times bigger than the fundamental period of the structure; therefore, it is necessary to install a flash memory (microSD format) with sufficient capacity, which is controlled by a power supply stage that deactivates it when not in use, thus saving energy. Finally, the CoreBoard incorporates the wireless communication stage that enables it to provide its services to the network layer of the IoT stack. For this purpose, the NB-IoT SIM7080G module from the manufacturer SIMCOM controlled by AT commands has been selected. This module exhibits consumption characteristics and functionalities that are highly positioned in the market. SensorBoard, depicted in Figure 2b, constitutes the other hardware component of the sensor system. It serves as the housing for the various sensors responsible for acquiring the physical measurements of the monitored structure. The key sensor is the triaxial accelerometer ADXL355 from Analog Devices with 20-bit (3.8 µg) resolution and ultra-low power consumption. This device has an embedded temperature sensor which is used to compensate for the accelerations taken in each case. In addition, the SensorBoard incorporates a Lantronix A2235-H GPS module with an integrated antenna, which is designed for low-power applications. It is a fundamental element for achieving time synchronization, with a low impact on the system autonomy [27], among the various nodes involved in monitoring structure, allowing subsequent OMA. Finally, the developed solution offers two extension interfaces that enhance adaptability for monitoring different components of a bridge (such as cable-stayed arch, deck, etc.) or bridges of various types. The first interface is analogue, designed to connect sensors such as strain gauges, and the second one is I2C, enabling the connection of digital sensors. After the design of the corresponding schematics and layout, the fabrication of both PCBs is carried out, which are integrated in an IP68 B140806ABS7035 enclosure together with a W3554B0140T PCB antenna and a 17Ah SAFT LSP 33600-20F battery. The overall cost for each node amounts to EUR 175.

**Figure 3:**
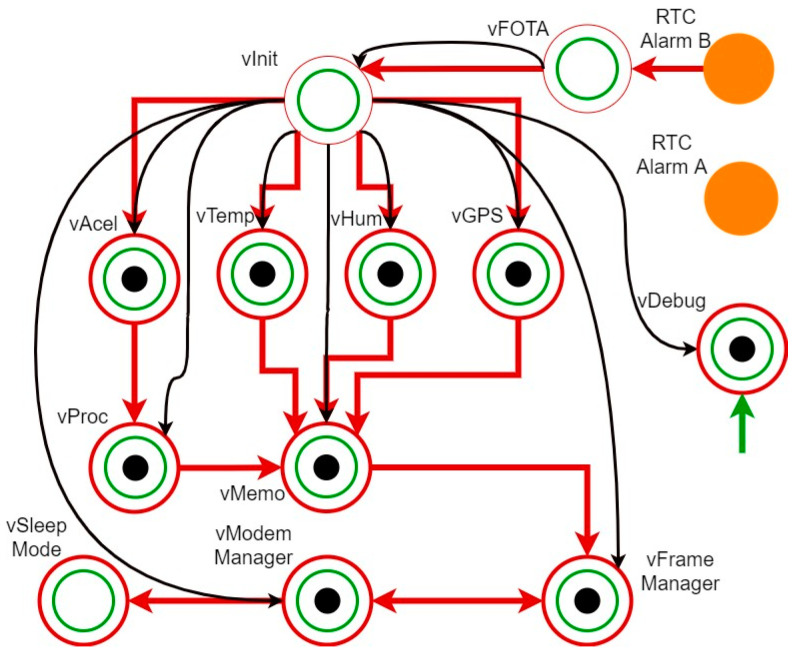
Firmware task architecture.

Firmware Design: Given the stringent timing requirements of the application and its inherent complexity, the firmware is designed according to the FreeRTOS task architecture shown in Figure 3. In this architecture, the red arrows represent the queues and the direction for sending data between tasks (red circles) and the black arrows represent the semaphores that control the execution of those tasks that have the black circle within them.

The functionality of each task is as follows:

- Real-Time Clock (RTC) Alarm A and RTC Alarm B: These tasks are associated with two native peripherals of the microcontroller that remain active during the Sleep Mode phase. The first one is a timer that, every 25 s, feeds the WatchDog Timer (WDT). RTC Alarm B is associated with the timer in charge of waking up the microprocessor and initiating Active Mode. This allows the microcontroller to restart in case it enters an unknown working state.
- vFOTA (Firmware Over The Air): Its functionality is to check if there is a new firmware version available for its installation. It is important to note that the node always retains the last properly functioning firmware version in its memory, so that if the new installation suffers any failure, the node can revert to the previous code version, avoiding the node’s disabling. This task is of vital importance in IoT applications, particularly in scenarios where deployments are extensive, geographically dispersed, and located in challenging access areas, both bureaucratically and physically. Without this capability, upgrading devices in the field would significantly increase maintenance costs, which is one of the key objectives of the presented development.
- vInit: Once the new firmware version has been installed or it has been checked that there is no new firmware version, this task is in charge of launching the measurement tasks of all the sensors installed in the node (through data queues), allowing them to acquire data concurrently. Additionally, it oversees launching the vMemo, vFrameManager and vModemManager tasks.
- vAcel, vTemp, vHum and vGPS: These are the tasks in charge of acquiring information from the monitored structure and managing the power consumption modes of each sensor. Each task is dedicated to a specific sensor and handles its activation, data acquisition, and control of energy consumption. From an architectural point of view, having a modular design with a task for each sensor provides enough versatility to activate or deactivate any sensor, or even add new sensors, without the need to modify the existing tasks. The vGPS task is especially important, as it acquires the GPS time signal, which is common to all deployed nodes, and assigns it to the internal clock of the microcontroller, ensuring the time synchronization between them while not having a significant impact on the power consumption of the whole node [27].
- vProc: This is the task in charge of processing the collected acceleration data before storing it in memory, which minimizes the memory depth required for storage. To achieve this, the task applies the RDT technique to the acceleration samples collected from the structure. The implementation and validation of this technique and its impact on node autonomy are detailed in Section 3.2. Although the task is presented with a single data input for clarity, it supports input from any sensing task, if necessary.
- vMemo: This task receives the data acquired by the sensing tasks or the results of the vProc task through data queues and stores them in the flash memory until they are successfully sent to the application layer.
- vFrameManager: This is the task in charge of reading data from the flash memory and organizing them into data frames to send to the central remote server.
- vModemManager: This task is responsible of establishing the NB-IoT connection (when the node is activated) and sending the data coming from vFrameManager through MQTT topic publications (Transmission Control Protocol—TCP) with an SSL security certificate.
- vSleepMode: The vSleepMode task plays a crucial role in optimizing power consumption. After successfully sending all data to the IoT application layer, this task activates the very-low-power mode. It suspends all the operating system tasks and gives the corresponding commands to the microprocessor to configure the RTC A and B alarms for the next operation cycle.
- vDebug: This task serves as a valuable tool for developers and users of the node. It receives and displays relevant information of all the tasks that are being executed in the microcontroller according to the verbose level established, on which the detail of the information displayed depends.

As said above, the developed firmware architecture is mainly focused on minimizing the energy consumption of the node. To this end, the complete working cycle, *t_cycle_* = *t_active_* + *t_sleep_*, is composed of the time in which all detailed tasks are performed (*t_active_*) and the time in which all devices are in a low-power state (*t_sleep_*) and complies with the relationship *t_sleep_ >> t_active_*, where *t_sleep_* is maximized as much as possible.

It is important to note that the monitoring nodes have been validated at both the firmware and hardware level in a laboratory test bench as well as in the field deployments described in Section 3 below.

#### 2.1.2. Network Layer

The network layer of the IoT stack oversees establishing a communication channel between the perception and application layers. For the presented solution, the NB-IoT technology has been selected as it is the one that best meets the specific requirements of the system [30]. This is because, compared to other standards used in the IoT, such as LoRaWAN, which always needs an additional connection interface to the cloud (3G, 4G, wired network, etc.) and offers a higher latency and more expensive deployments, it is an operational network, which is operated, standardized (3GPP standard) and growing [31], that offers coverage to users at a very low cost (around EUR 1/year in Spain in 2024, for the amount of data required [32]). This avoids high maintenance costs, especially in the management of geographically distributed structures, where it is necessary to deploy as many networks as structures. On the other hand, NB-IoT directly allows the application of the MQTT protocol, which is the most widely used in the IoT framework, mainly because it is both lightweight and secure as a publish–subscribe protocol and it is oriented to low-bandwidth applications and limited-memory devices [33].

#### 2.1.3. Cloud Application Server

**Figure 4:**
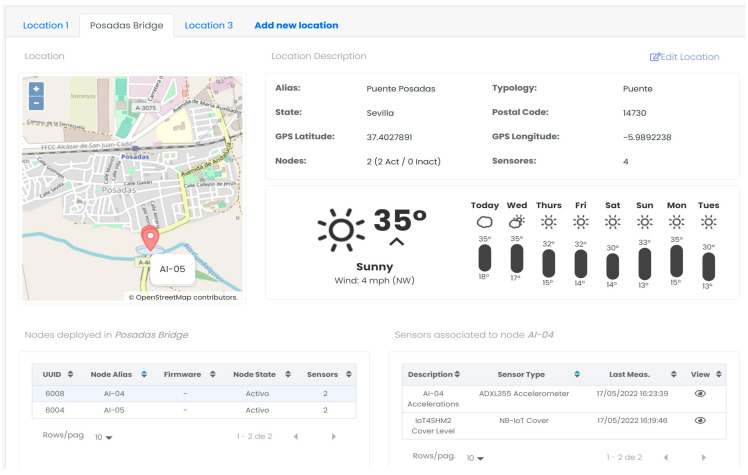
Graphical user interface. Main view.

**Figure 5:**
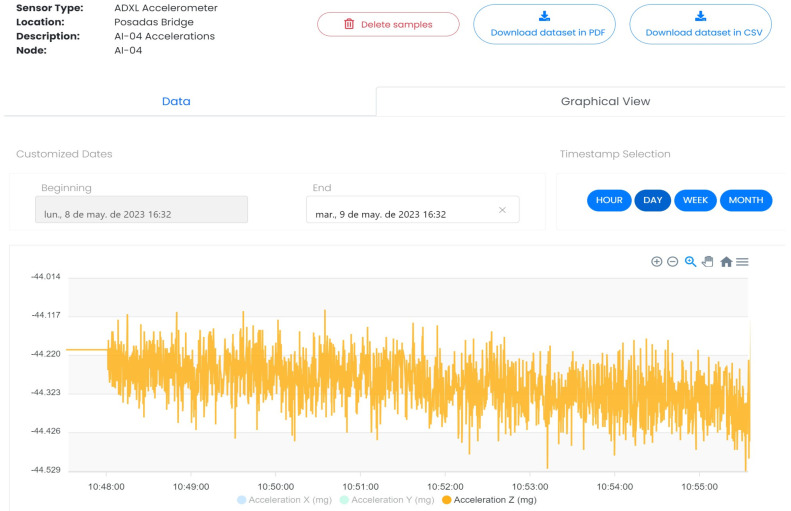
Graphical user interface. Acceleration view.

As mentioned in Section 2, the application server is hosted in the cloud and is responsible for exploiting the information received from the nodes through the 3 layers of perception, network and application. Its primary role is to leverage this information and provide end users with a comprehensive solution through various tools for managing the monitored assets. The application server offers numerical and graphical representations of the data, enabling users to visualize and analyze the information in a meaningful way. Additionally, it provides relevant mechanisms for remotely updating the firmware of the nodes, if required. To achieve this goal, the server is made up of a set of microservices deployed in Docker application containers, which are described below, and which communicate with each other through the database:

- Database: This is the element that stores all the system’s information, both that sent by the nodes and that generated by the high-level applications, such as the firmware versions of each of the nodes. This implies the storage of highly heterogeneous information, which makes it necessary to use a non-relational database technology.
- Decoder: This is the microservice responsible for decoding the information received by the nodes. This decoding is carried out according to the common data model known by both the vFrameManager of the perception layer and the decoder of the cloud application server. At this point, the authenticity of the information is also checked, as well as the validity of the information, considering its own encoding and the CRC included in the useful data.
- Encoder: The main functionality of this service is the encoding of the information sent downlink to the devices, primarily focused on firmware updates for the nodes. In the same way as in the uplink, the information sent (in this case a binary file) is encoded according to the data model known by both the encoder and the vFota task.
- Processing: This is the service in charge of transforming and leveraging the valuable information received from the nodes. In the presented solution, this service focuses on analyzing the data acquired by the deployed accelerometers and extracting the natural frequencies of the monitored structures using the Stochastic Subspace Identification (SSI) methodology [34]. Additionally, it is responsible of processing the rest of the information coming from the nodes, such as the meteorological operating conditions of the structure, from which the limit working conditions to which the structure is subjected are extracted. The aim is to incorporate these conditions into the calculation of the structure’s useful life, enabling a more accurate assessment of its durability and performance over time.
- Graphical User Interface: The user interface, shown in Figure 4 and Figure 5, has been developed based on a series of web views using JavaScript as the programming language as well as to register all the actions performed by all the services and users and to guarantee their traceability. The services offered by the GUI are the following:
  ○ A scheme of access and/or editing permissions applicable to the different users that guarantees the security of the data stored and offers the user only the information and services available according to their role, which, given that these are strategic structures, is of vital importance.
  ○ Detailed management (editing, creation, deletion, etc.) of both the monitored structures and the deployed nodes, as well as the sensors available and the information collected by each of them. This allows the geopositioning of each node in map view (Figure 4) and the presentation of the information from the different sensors in both numerical and graphical table mode (Figure 5).
  ○ The processing block makes it possible to exploit the information collected by the nodes. In the presented application, this entails calculating and monitoring the fundamental frequencies of monitored structures. It also involves managing alarms through email, SMS, or visual notifications on the interface when these frequencies deviate beyond a configurable threshold, indicating potential damage to the monitored structure. Additionally, this block combines the obtained results with data acquired from official government seismographs, providing an integrated response that adds value to the system and aligns with the state-of-the-art practises.
  ○ It also offers an interface for exporting and importing data, which potentially allows its integration with other market monitoring solutions.

At this point, it is important to note that the implementation of the complete solution, from the lowest-level hardware to the highest-level software, would allow, at more advanced stages of technological maturity, for adaptations to the solution in general and, more specifically, the graphical interface to the demands of potential users.

Additionally, as can be seen, it is a modular design that allows the scalability of the system, both in terms of devices and their functionalities or value-added services at the cloud level, regardless of the number of deployed nodes, thanks to the use of the communication networks operated.

## 3. Results

The validation of the developed system consists of three different experiments:

1. Characterization of the node’s power consumption and dispatch times (Section 3.1) by quantifying the impact of the RDT application (Section 3.2);
2. Performance comparison and validation against a high-precision reference hardware and software system (Commercial System 1) in a pilot structure (Section 3.3);
3. Validation against another high-performance commercial hardware and software system (Commercial System 2) at the Eduardo Torroja bridge in Posadas (Córdoba, Spain) (Section 3.4).

### 3.1. Sensor Node Characterization: Power Consumption

**Figure 6:**
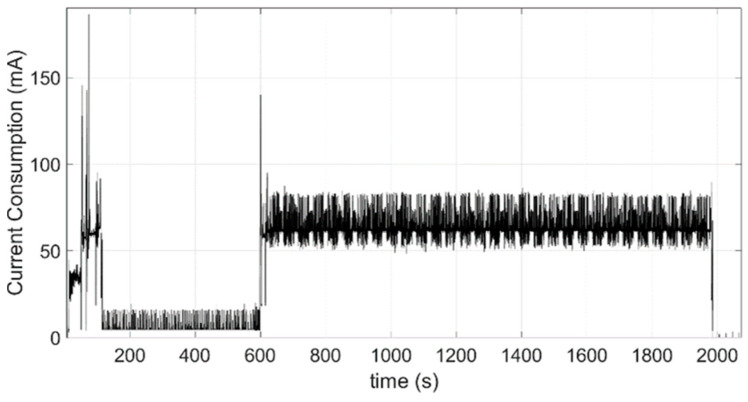
Current consumption of a working operation cycle.

As justified in Section 2.1.1, the acceleration data series should be greater than 1000 times the fundamental period of the monitored structure. Considering that the first fundamental frequency of this type of structure is in the range 1–3 Hz, the length of the data series should be about 8 min. Additionally, considering that the sampling frequency must be at least twice the highest frequency (Nyquist sampling theorem) and that the frequencies of structural interest are not above 10 Hz, the chosen sampling frequency of 31.25 Hz is the lowest available frequency in the selected accelerometer that satisfies the Nyquist criteria. This implies taking a total of 15,000 samples of 20-bit resolution from each axis. This process results in a work cycle with power consumption. As can be seen, Figure 6 illustrates the energy consumption of a node in the active state, monitored using a Keithley 2450 SourceMeter. It can be divided into the following main stages:

- GPS connection: An average consumption of 31.56 mA for 40.77 s;
- Wireless network connection: This includes SSL authentication and has an average consumption of 59.46 mA for 64.07 s;
- Data acquisition: An average consumption of 4.77 mA for 480 s;
- Data transmission: An average consumption of 63.09 mA for 1383.91 s.

In total, these stages result in a consumption of 26.33 mAh per operation cycle. In the idle state, the node has a deep sleep consumption of 5 µA. Additionally, there is a periodic consumption of 1 mA for 0.25 s each time the WDT feeding task is activated, which occurs every 25 s. Therefore, assuming daily periodic monitoring [35], which could be carried out at different times of the day to obtain the structure characterization under various operating conditions, and with a battery capacity of 17,000 mAh, the node has an autonomy of 638 days. One of the key findings from the previous study is that Figure 5 highlights the significant impact of wireless transmission of a large volume of information on node consumption, indicating that minimizing this data volume can maximize node lifetime. For this reason, taking advantage of the fact that the processing capabilities of the microcontroller allow the deployment of embedded edge computing algorithms, Section 3.2 proposes and validates the use of RDT as a methodology for reducing the information to be transmitted wirelessly by the nodes.

### 3.2. RDT Application to IoT-SHM: Implementation and Quantifying Power Consumption Saving

**Figure 7:**
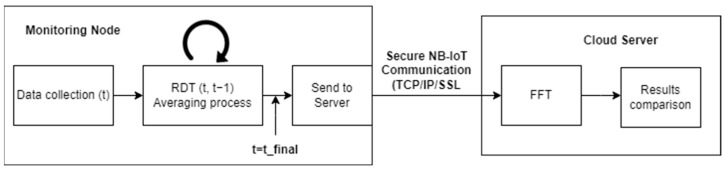
Data flow.

The Random Decrement Technique (RDT) offers a significant advantage in reducing the volume of information that needs to be transmitted wirelessly by the nodes. The technique involves dividing the signal into multiple segments based on a trigger condition. These segments are then merged into a single segment by calculating the arithmetic mean. This is supported in [36], where it is demonstrated how if the RDT is applied over a zero-mean stationary Gaussian stochastic process, the results are analogous to the original signal and its dynamic properties. This technique was first used by Cole [37] in 1968, whose objective was to analyze the dynamic response of spatial structures subjected to environmental loads. Since then, this technique has found applications in various areas of structural analysis, including mode identification in non-linear [38] and non-stationary environments [39], and general acquisition of structural characteristics [40,41]. Therefore, these applications demonstrate the versatility of RDT in obtaining modal parameters and monitoring structural health. In the context of this work, RDT is applicable for reducing data transmission and thus ensuring longer battery life because the suggested system uses an IoT architecture with autonomous monitoring nodes. To effectively apply the RDT, it is necessary to define two essential parameters for each monitored structure: the number of acceleration samples to be acquired and the signal trigger level for collecting the signals. To provide insights into how to determine the appropriate values for these parameters, the calculation procedure for the pilot structure and Eduardo Torroja bridge (Spain) (detailed in Section 3.3 and Section 3.4, respectively) will be described. To achieve this, a real dataset of 15,000 samples of accelerations will be obtained and the Fast Fourier Transform (FFT) will be applied to the dataset at the central server, resulting in a reference set of natural frequencies for the specific structural point. Subsequently, following the data flow shown in Figure 7, the RDT technique will be locally applied at the monitoring nodes (through the vProc firmware task) to the complete set of samples. The threshold and the number of samples in the resulting segments will be varied during the analysis. The summed segments will then be wirelessly transmitted to the central server. At the server, an FFT algorithm will be applied to the received data to compare the results with the FFT performed on the complete set of accelerations. The objective is to identify the case where the natural frequencies are obtained with high precision while minimizing computational and resource costs.

**Figure 8:**
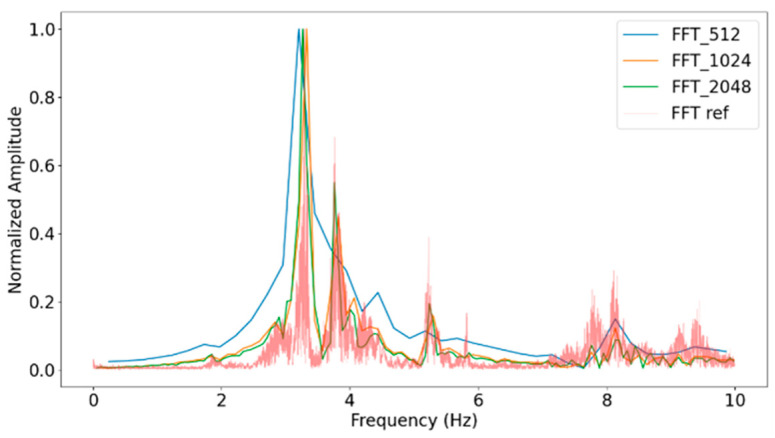
Fast Fourier Transform for different numbers of samples.

The analysis is conducted using different numbers of acceleration samples, specifically 256, 512, 1024 and 2048, as shown in Figure 8 and Table 1. According to [39], the trigger level is set to $\sqrt{2}\sigma$, where σ represents the standard deviation.

**Figure 9:**
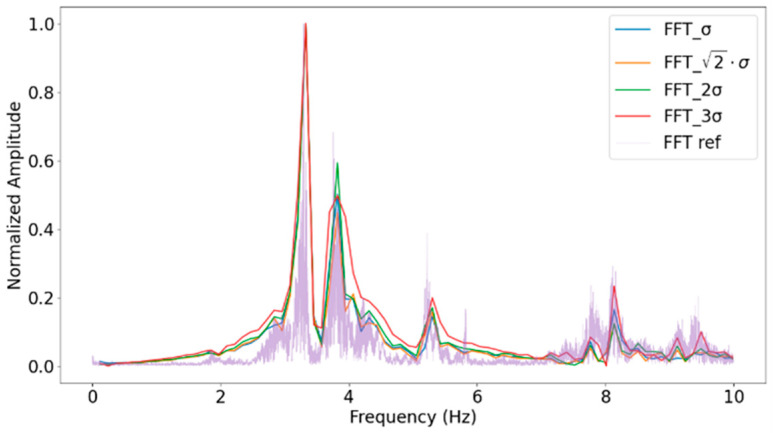
Fast Fourier Transform for different trigger levels.

Furthermore, according to [42], the recommended value for the trigger level is $\sqrt{2}\sigma$. However, to verify the effectiveness of this parameter in detecting natural frequencies, the trigger level value has been tested and its behaviour is illustrated in Figure 9 and Table 2.

The results obtained in Table 2 confirm that the optimal value for the trigger level is $\sqrt{2}\sigma$, with 1972 segments, since the error in obtaining the modes is the lowest. Lower threshold values result in more segments (3064) and less accurate results (up to 13.614% error), as they include more noise. While values above this threshold (2σ and 3σ) may lead to the loss resolution of some structural modes (mode 4) due to a smaller number of segments (1196 and 685, respectively). Therefore, the validation supports the use of a trigger level of $\sqrt{2}\sigma$.

When it comes to deciding the threshold, one might think that the resources used to store the different segments in memory could be an important factor. However, it is worth highlighting that there are no limitations in the allocation of local segments on the sensor node since the segment mean is calculated in the microprocessor on the fly (allocation of all datasets acquired is not necessary) by aggregating the individual segments into a 32-bit array of a sample size of 1024 (4 kB in RAM with the ability to store up to one million segments) and then averaging with the number of segments collected.

Therefore, values of $\sqrt{2}\sigma$ for the trigger level and 1024 for the segment sample size are selected. Next subsection (Power Consumption Analysis) evaluates the impact of this configuration over the nodes’ power consumption and over its autonomy.

**Figure 10:**
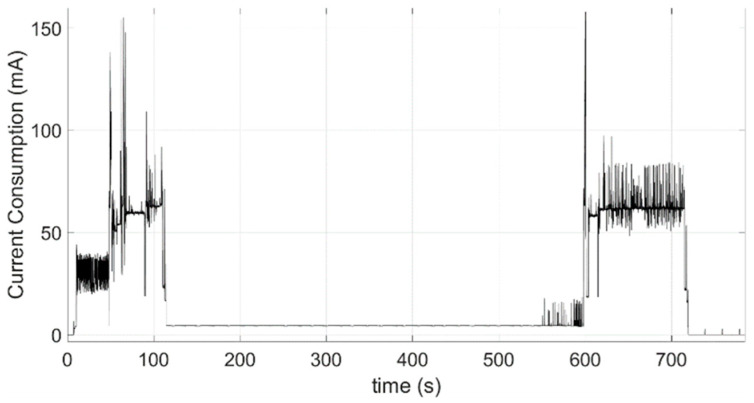
Current consumption of a working cycle with RDT implementation.

*Power Consumption Analysis*: Once a segment size of 1024 samples and a threshold of $\sqrt{2}\sigma$ have been selected for the Eduardo Torroja bridge’s characterization, the current consumption profile is shown in Figure 10. This profile closely resembles the one shown in Figure 6, with two key differences. The first and most important is the reduction in the information transmission time to 117 s, resulting in saving about 22 mAh per operation cycle. The second difference, although it does not have a high impact on power consumption (saving 0.04 mAh), is an important functional difference. Figure 9 shows how the current peaks of up to 16 mA that are present in Figure 5 during acquisition time disappear. It is because the implementation of RDT and its work on the fly avoids the use of a microSD card until the last moments of this phase, when the results of RDT functions are stored. Under these conditions, considering the same energy consumption in the idle phase and daily monitoring and the same battery model, an autonomy of 3.718 days, i.e., more than 10 years, can be achieved.

The functional characterization of the RDT presented in this section, along with its significant energy saving impact in SHM, validate the application of this technique in the field of structural monitoring, enabling unattended deployment. Therefore, the next phase involves conducting an experimental validation in the field on two different structures.

### 3.3. Pilot Structure Test

**Figure 11:**
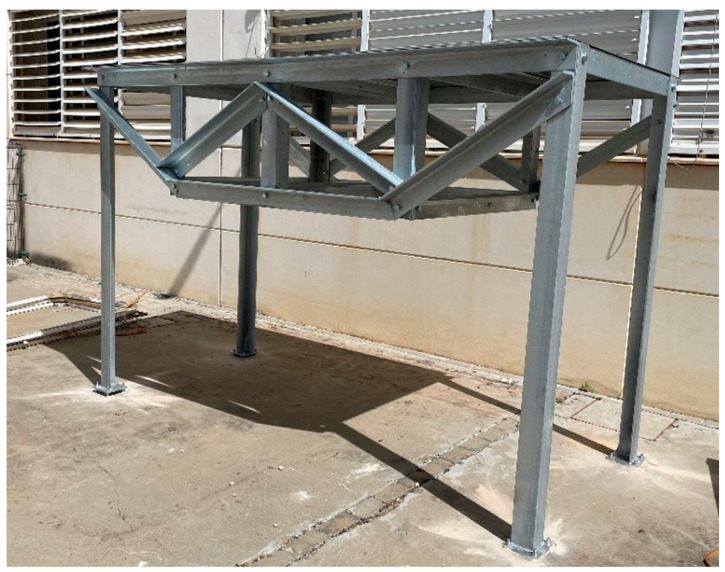
Pilot structure.

**Figure 12:**
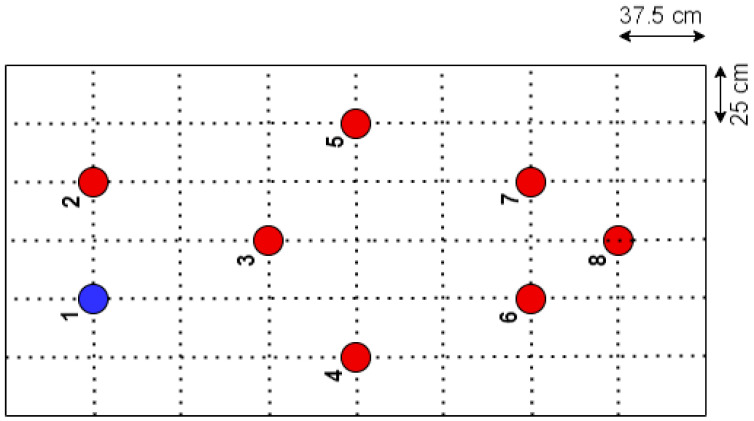
Sensor deployment diagram.

**Figure 13:**
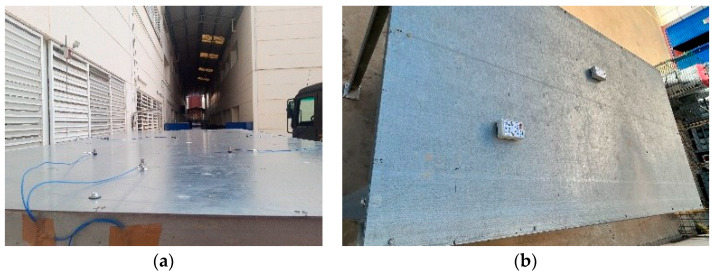
Sensor allocation: (**a**) reference system deployment; (**b**) the system deployment of this study. One case.

**Figure 14:**
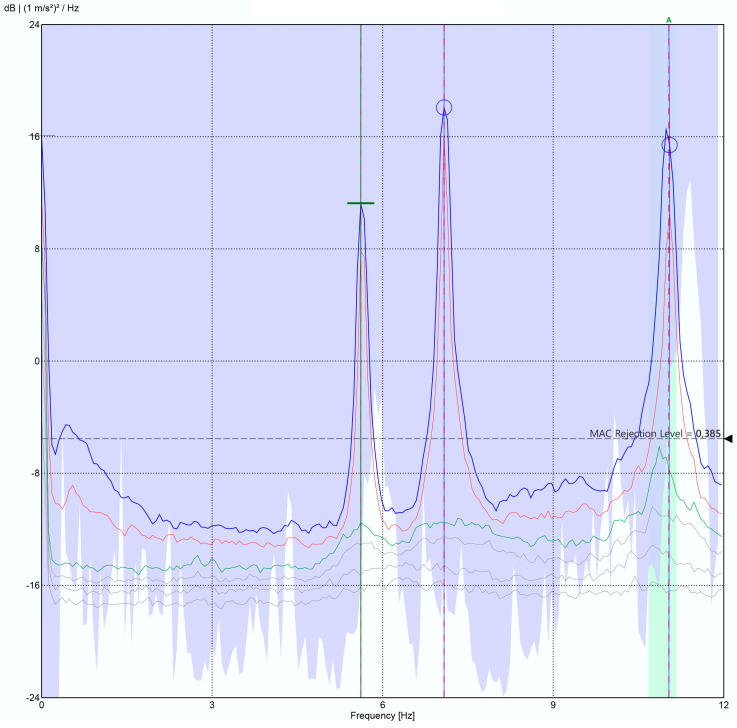
Pilot structure response: commercial reference system.

The initial testing of the developed solution was conducted on a metallic structure of galvanized steel of quality S-275JR, with a floor area of 3 × 1.5 m^2^ and a height of 2 m (Figure 11), whose structure is inspired by the bridge of Eduardo Torroja, located in the town of Posadas (Córdoba), and has been manufactured with annular profiles of 100 × 10 mm^2^ and assembled in the facilities of the School of Engineering of the University of Seville. The characterization of the structure was carried out with two monitoring systems: a commercial precision system (Commercial System 1) and the solution presented in this work. Commercial System 1 consists of eight PCB Piezotronics 352C33 analogue accelerometers with a sensitivity of 100 mV/g, an eight-channel SIRIUS-8xACC data acquisition system with 24-bit analogue-to-digital converters, and the Dewesoft X-Structural-Analyzer software package (version 2020.2), valued at a total of EUR 11500. In order to carry out the tests, a division was made in elements of the structure board as shown in Figure 12, placing the eight commercial accelerometers in the indicated positions and taking the one in position 1 as the reference (Figure 13a,b). The excitation was a low-frequency signal (lower than 2 kHz) generated by using a Roving Hammer. These data, once processed in the ARTEMIS v7.2 structural analysis software, gave the results shown in Figure 14 and Table 3.

**Figure 15:**
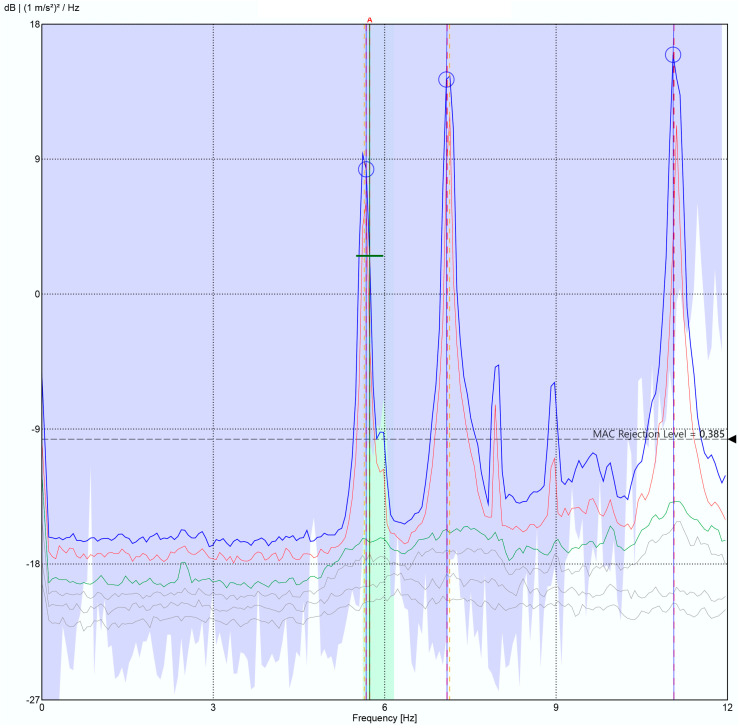
Pilot structure response: the system of this study.

In the case of the developed solution, following the traditional methodology [36] with RDT parameters calculated for this structure ($\sqrt{2}\sigma$ for the trigger level and 1024 segment samples), a total of seven tests were performed using two nodes. One node was fixed at the reference position, position 1, while the other node was placed at positions 2, 3, 4, 5, 6, 7, and 8 for each test. These nodes were configured, as detailed in Section 3.1., to sample time series of 15,000 triaxial samples at 31.25 Hz. Subsequently, the collected data, once sent to the application server, were processed with the ARTEMIS v7.2 software, yielding the results shown in Figure 15 and Table 3.

The data shown in Table 3 demonstrate very similar natural frequencies, with a maximum deviation of 1.05% in the first vibration modes of the monitored structure, which are those with more structural significance, which results in sufficient accuracy [43,44,45]. Therefore, these results validate the use of this solution for the intended application.

### 3.4. Eduardo Torroja Bridge Test

**Figure 16:**
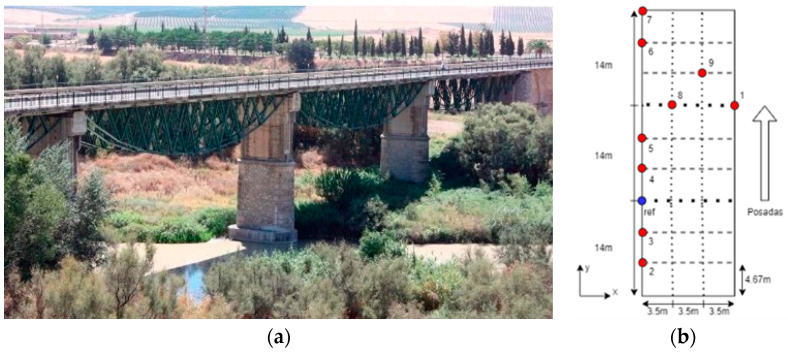
Field test configuration: (**a**) Eduardo Torroja bridge; (**b**) sensor deployment.

This bridge (Figure 16a), built by Eduardo Torroja, a renowned figure in Spanish civil engineering, belongs to the cultural heritage of Andalusia (Spain). It features a distinctive design, comprising a combination of a concrete deck and 6 m high inverted parabolic arch trusses. As a result of damage suffered during the Spanish Civil War, the bridge was redesigned as a five-span structure of 43 m and 11 m wide. This bridge is of particular interest as, due to its unique geometry, the parabolic steel structure is under the main deck and is therefore not accessible for structural monitoring and characterization, and therefore, the unattended and low-cost solution proposed in this work is of relevance.

**Figure 17:**
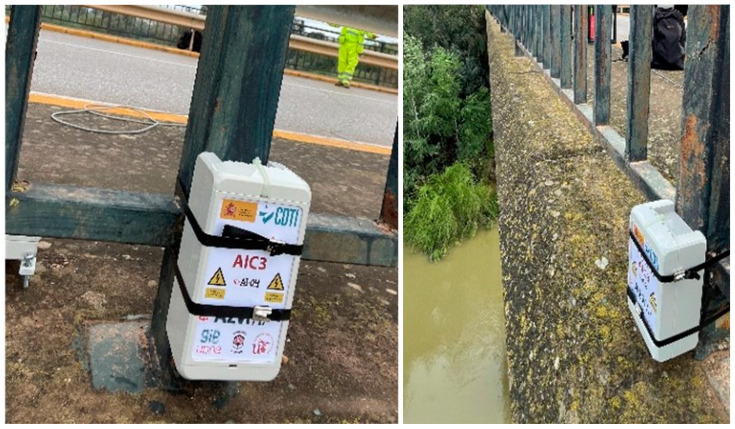
Sensor node deployment.

The testing procedure employed for the system validation aligns with the methodology outlined in Section 3.3, with a segment size of 1024 samples and a threshold of $\sqrt{2}\sigma ,$ estimated in Section 3.2. The configuration for these tests involved acquiring 15,000 samples at a frequency of 31.25 Hz. In this case, two nodes were used, a reference node located at the “ref” position in Figure 16b and another located at locations 1 to 9 successively. These nodes were strategically positioned to monitor the bridge deck, with measurement points 1–7 situated on the fence (Figure 17) and measurement points 8 and 9 located directly on the bridge deck.

**Figure 18:**
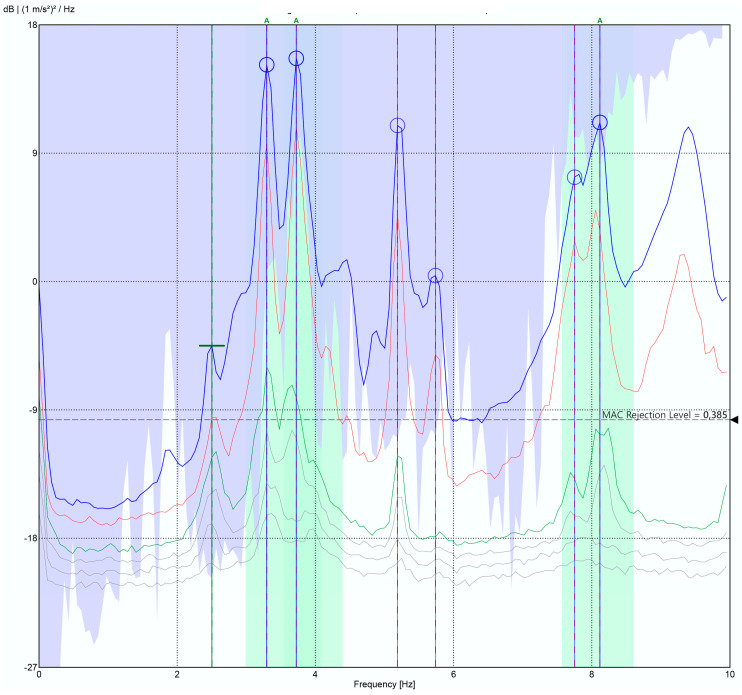
Structural response acquired by this work’s system.

As can be seen in Figure 18, the Power Spectral Density (PSD), obtained in ARTEMIS v7.2 software, clearly shows the fundamental frequencies of the bridge in the range of interest (3 Hz to 10 Hz), which are located at the frequencies detailed in Table 4. For the validation of the obtained results, these are compared in Table 4 with those obtained by [28], which presents a detailed characterization of this bridge through a battery of monitoring tests, carried out under the same methodology, in March 2017. For this purpose, [28] utilized four high-precision GMSplus accelerometers (triaxial monitoring, a bandwidth of up to 250 Hz and a sensitivity of 10 V/g) called Commercial System 2. The tests included various setups with environmental excitation induced by wind and traffic.

As can be seen in Table 4, the developed system presents a maximum deviation of 1.72% compared to the results from [43]. This deviation falls within an acceptable range for the intended application, as justified in Section 3.2. It is worth noting that our system does not detect modes 3 and 5, which is due to its proximity to modes 2 and 6 (0.12 Hz and 0.03 Hz), respectively, and to the fact that the number of setup measurements is lower than that presented in [28] (9 vs. 12), as well as the fact that they were performed in different locations, alongside the different weather conditions, which are impossible to reproduce in structures of this size. Nevertheless, the provided results from both the pilot structure and the Eduardo Torroja bridge offer a realistic and accurate representation of the structural behaviour of the monitored constructions. Consequently, performance of the developed system can be considered validated.

## 4. Discussion

From a technical perspective, it is evident from the comparison presented in Table 5 that both Commercial System 1 and Commercial System 2 offer higher-resolution performance. This is because these types of systems are specifically designed for monitoring various types of structures whose signal amplitude and modal responses may be very different, which may imply stricter time synchronization requirements. However, as demonstrated, the developed system offers an accuracy in the detection of modal frequencies higher than 1.72%. Additionally, it offers significant cost savings (two orders of magnitude, which generates a sufficiently significant margin to opt for a competitive commercial solution in terms of price) compared to other systems, providing added-value services such as unattended deployment and wireless and permanent installation capabilities in structures. Furthermore, it supports daily periodic monitoring and boasts an autonomy of over 10 years.

Additionally, the presented work includes a remote platform that enables the management of monitored structures and the exploitation of the gathered information through their modal characteristics. It also facilitates the implementation of new value-added services, thanks to its microservice-based architecture. Therefore, the presented system offers a novel and complete IoT solution, which meets the needs of each of the layers of the paradigm and offers valuable results to its users.

Therefore, the solution presented presents a technological maturity level of field validation, i.e., TRL7, and its next challenges are those focused on increasing the technological maturity level to a commercial product level (TRL9), which includes market studies, certifications of radio emissions, metrology, or access to the electromagnetic environment, among others.

## 5. Conclusions

This paper introduces a comprehensive SHM system designed for the identification of structural damage. The system is built upon an IoT framework, enabling temporally synchronous, low-cost, and low-power deployment for unattended monitoring. By leveraging edge processing capabilities and embedded RDT implementation, the system achieves an autonomy of over 10 years for the monitoring nodes. Furthermore, this development is complemented by the IoT server architecture deployed for efficient and secure management and control of the devices deployed in the field. This paper provides a detailed description of the complete reference architecture designed for this purpose. The perception layer of the architecture focuses on the modular hardware design of the IoT terminal node, divided into two boards called CoreBoard and SensorBoard. The CoreBoard is responsible for managing common resources such as power, communications and memory. It also controls the SensorBoard, focused on bridge monitoring thanks to the integration of sensors such as a high-performance MEMS accelerometer or GPS receiver. It also details the firmware deployed according to a FreeRTOS task architecture and a secure communication stack (SSL/MQTT/TCP/IP) and the integration of these with the hardware and the rest of the peripheral elements (battery and antenna) to form the final node. The network layer of the architecture justifies the selection of NB-IoT as the most suitable technology, compared to other IoT networks like LoRaWAN. Furthermore, a cloud application server has been implemented, utilizing containerized microservices. These microservices handle various tasks such as processing, the calculation of fundamental frequencies of the monitored bridges, and graphical representation of the information. This server provides an integrated tool for end users to efficiently manage the monitored structures. Subsequently, the energy consumption of the node was characterized, revealing that wireless data transmission has the most significant impact. To reduce power consumption, the RDT technique was implemented and validated, leveraging the edge computing capability of the node. As a result, the node’s autonomy was extended from 638 days to 3718 days. Finally, the system was validated in two steps: first by comparing its performance with Commercial System 1 through the monitoring of a scale bridge structure, and then with Commercial System 2 through a battery of tests on the Eduardo Torroja bridge. The results show how the proposed system, which costs two orders of magnitude less than the commercial ones, achieves an accuracy higher than 1.72% in detecting vibration modes. Its ability to be installed and operated unattended and periodically, coupled with its low cost and power consumption, positions the presented work as a novel solution for structural damage monitoring and detection with a Technology Readiness Level of 7, which is the starting point for the development of a certification plan prior to turning the presented work into a possible market solution.

## Figures and Tables

**Table 1:** Fast Fourier Transform for different numbers of samples.

Ref		FFT-512		FFT-1024		FFT-2048	
	Hz	Hz	Error (%)	Hz	Error (%)	Hz	Error (%)
Mode 1	3.32	3.32	0	3.32	0.24	3.26	1.59
Mode 2	3.82	-	-	3.82	0.05	3.76	3.14
Mode 3	5.42	5.67	4.63	5.54	2.36	5.54	2.25
Mode 4	5.91	-	-	5917	0.11	5.85	0.91
Mode 5	7.15	7.15	0.12	7.15	0.12	7.21	0.86
Mode 6	7.76	-	-	7.76	0.09	7.76	0.09
Mode 7	8.13	8.13	0.07	8.13	0	8.13	0.07

**Table 2:** Natural frequencies obtained from different trigger levels.

Ref		FFT-σ		FFT-$\sqrt{2}\sigma$		FFT-2$\sqrt{2}\sigma$	
	Hz	Hz	Error (%)	Hz	Error (%)	Hz	Error (%)
Mode 1	3.32	3.77	13.61	3.32	0.24	3.32	0.24
Mode 2	3.82	4.00	4.89	3.82	0.05	3.82	0.05
Mode 3	5.42	5.20	4.00	5.54	2.36	5.54	2.36
Mode 4	5.91	5.84	1.13	5.91	0.11	6.41	8.47
Mode 5	7.15	-	-	7.15	0.12	7.15	0.12
Mode 6	7.76	7.91	1.98	7.76	0.09	7.76	0.09
Mode 7	8.13	8.2	0.86	8.13	0	8.13	0.86

**Table 3:** Pilot structure response: comparison of results.

	Commercial System (Hz)	This Work (Hz)	Difference (%)
Mode 1	5617	5676	1.05
Mode 2	708	7091	0.155
Mode 3	11,035	11,062	0.244

**Table 4:** Fundamental frequency comparison.

Modes	Reference System (Hz) [28]	This Work (Hz)	Difference (%)
Mode 1	3.28	3296	0.48
Mode 2	3.68	3723	1.17
Mode 3	3.80	-	-
Mode 4	5.14	5188	0.93
Mode 5	5.61	-	-
Mode 6	5.64	5737	1.72
Mode 7	7.79	7751	0.5
Mode 8	8.02	8118	1.22

**Table 5:** System comparison.

Feature	Commercial System: Pilot Structure	Commercial System: Eduardo Torroja Bridge	This Work
Accelerometer	352C33	GMSplus	ADXL355
Acquisition System	SIRIUS-8xACC	GMSplus	This work
Accelerometer accuracy	24b ϵ − ∆ ADC	24b ϵ − ∆ ADC	20 bits
Power Supply (Vdc)	9–36	12.5–18	3.6
Autonomy (years)	-	-	10 (once a day)
Management Platform	No	No	Yes
Price	EUR 12,200	EUR 8000/unit	EUR 175/unit

## Data Availability

Data are contained within the article.

## References

[1] Farrar C.R., Worden K. (2012). Structural Health Monitoring: A Machine Learning Perspective.

[2] Farrar C.R., Worden K. (2007). An introduction to structural health monitoring. Philos. Trans. R. Soc. A.

[3] Richardson M.H. (1980). Detection of Damage in Structures from Changes in Their Dynamic (Modal) Properties, a Survey.

[4] Pepi C., Gioffré M., Comanducci G., Cavalagli N., Bonaca A., Ubertini F. (2017). Dynamic characterization of a severely damaged historic masonry bridge. Procedia Eng..

[5] Conde B., Ramos L.F., Oliveira D.V., Riveiro B., Solla M. (2017). Structural assessment of masonry arch bridges by combination of non-destructive testing techniques and three-dimensional numerical modelling: Application to Vilanova bridge. Eng. Struct..

[6] Lynch J.P. (2006). A Summary Review of Wireless Sensors and Sensor Networks for Structural Health Monitoring. Shock Vib. Dig..

[7] Hamburger R.O. (2000). A Policy Guide to Steel Moment-Frame Construction.

[8] Cunha A., Caetano E. (2006). Experimental Modal Analysis of Civil Engineering Structures. Sound Vib..

[9] Salehi H., Burgueño R., Chakrabartty S., Lajnef N., Alavi A.H. (2021). A comprehensive review of self-powered sensors in civil infrastructure: State-of-the-art and future research trends. Eng. Struct. /Eng. Struct. (Online).

[10] Lynch J.P. (2007). An Overview of Wireless Structural Health Monitoring for Civil Structures. Philos. Trans. Math. Phys. Eng. Sci..

[11] Ha D.H., Park H.S., Choi W.G., Kim Y. (2013). A Wireless MEMS-Based Inclinometer Sensor Node for Structural Health Monitoring. Sensors.

[12] Haus J.N., Lang W., Roloff T., Rittmeier L., Bornemann S., Sinapius M., Dietzel A. (2022). MEMS Vibrometer for Structural Health Monitoring Using Guided Ultrasonic Waves. Sensors.

[13] Shang B.-L., Song B.-F., Chang F. New Sensor Technologies in Aircraft Structural Health Monitoring. Proceedings of the 2008 International Conference on Condition Monitoring and Diagnosis.

[14] Hassani S., Dackermann U. (2023). A Systematic Review of Advanced Sensor Technologies for Non-Destructive Testing and Structural Health Monitoring. Sensors.

[15] Sabato A.F., Feng M.Q., Fukuda Y., Carní D.L., Fortino G. (2016). A Novel Wireless Accelerometer Board for Measuring Low-Frequency and Low-Amplitude Structural Vibration. IEEE Sens. J..

[16] Yu Y., Ou J., Zhang J., Zhang C., Li L. (2009). Development of Wireless MEMS Inclination Sensor System for Swing Monitoring of Large-Scale Hook Structures. IEEE Trans. Ind. Electron..

[17] Wang M., Koo K., Liu C., Fei X. (2023). Development of a low-cost vision-based real-time displacement system using Raspberry Pi. Eng. Struct./Eng. Struct. (Online).

[18] Wang H., Guo J., Mo H., Zhou X., Han Y. (2023). Fiber Optic Sensing Technology and Vision Sensing Technology for Structural Health Monitoring. Sensors.

[19] Gomez J., Zubia J., Aranguren G., Durana G., Illaro J.A., Saez I., Kirchhof M., Poisel H., Hartl E. Comparing Polymer Optical Fiber (POF), fiber Bragg gratings and traditional strain gauge for aircraft structural health monitoring. Proceedings of the 2008 IEEE Avionics, Fiber-Optics and Photonics Technology Conference.

[20] Da Costa Antunes P., Lima H.F., Alberto N., Rodrigues H., Pinto P., De Lemos Pinto J., Nogueira R.N., Varum H., Costa A., De Brito Andre P.S. (2009). Optical Fiber Accelerometer System for Structural Dynamic Monitoring. IEEE Sens. J..

[21] Pham Q., Ta Q., Park J., Kim J. (2022). Raspberry Pi Platform Wireless Sensor Node for Low-Frequency Impedance Responses of PZT Interface. Sensors.

[22] Sapidis G., Naoum M., Papadopoulos N., Voutetaki M. (2023). Flexural Damage Evaluation in Fiber Reinforced Concrete Beams Using a PZT-Based Health Monitoring System. International RILEM Conference on Synergising Expertise towards Sustainability and Robustness of CBMs and Concrete Structures.

[23] Tokognon C.A., Gao B., Tian G.Y., Yan Y. (2017). Structural Health Monitoring Framework Based on Internet of Things: A Survey. IEEE Internet Things J..

[24] Mahmud M.A., Bates K., Wood T., Abdelgawad A., Yelamarthi K. A complete Internet of Things (IoT) platform for Structural Health Monitoring (SHM). Proceedings of the 2018 IEEE 4th World Forum on Internet of Things (WF-IoT).

[25] Montori F., Zyrianoff I., Gigli L., Calvio A., Venanzi R., Sindaco S., Sciullo L., Zonzini F., Zauli M., Testoni N. (2023). An IoT Toolchain Architecture for Planning, Running and Managing a Complete Condition Monitoring Scenario. IEEE Access.

[26] Bisio I., Garibotto C., Lavagetto F., Sciarrone A. A Novel IoT-based Edge Sensing Platform for Structure Health Monitoring. Proceedings of the IEEE INFOCOM 2022-IEEE Conference on Computer Communications Workshops (INFOCOM WKSHPS).

[27] Fort E.H., Blanco-Carmona P., Garcia-Oya J.R., Chavero F.M., Carvajal R.G., Serrano-Chacon A.R., Mascort-Albea E.J. (2023). Wireless and Low-Power System for Synchronous and Real-Time Structural-Damage Assessment. IEEE Sens. J..

[28] Pachón P., Castro R.L., García-Macías E., Compán V., Puertas E.E. (2018). Torroja’s bridge: Tailored experimental setup for SHM of a historical bridge with a reduced number of sensors. Eng. Struct..

[29] Cantieni R. Experimental methods used in system identification of civil engineering structures. Proceedings of the International Operational Modal Analysis Conference (IOMAC).

[30] Iqbal M., Abdullah A.Y.M., Shabnam F. An Application Based Comparative Study of LPWAN Technologies for IoT Environment. Proceedings of the 2020 IEEE Region 10 Symposium (TENSYMP).

[31] Stanco G., Botta A., Frattini F., Giordano U., Ventre G. On the performance of IoT LPWAN technologies: The case of Sigfox, LoRaWAN and NB-IoT. Proceedings of the ICC 2022-IEEE International Conference on Communications.

[32] 1NCE Precios. (May 2023). 1NCE. https://1nce.com/es-es/1nce-connect/10-euros-por-10-anos.

[33] Swamy S.N., Jadhav D., Kulkarni N. Security threats in the application layer in IOT applications. Proceedings of the 2017 International Conference on I-SMAC (IoT in Social, Mobile, Analytics and Cloud) (I-SMAC).

[34] Peeters B., De Roeck G. (2001). Stochastic System Identification for Operational Modal Analysis: A Review. J. Dyn. Syst. Meas. Control-Trans. ASME.

[35] Carpinteri A., Lacidogna G., Pugno N.M. (2007). Structural damage diagnosis and life-time assessment by acoustic emission monitoring. Eng. Fract. Mech..

[36] Rodrigues J., Brincker R., Brincker R., Møller N. (2005). Application of the Random Decrement Technique in Operational Modal Analysis. Proceedings of the 1st International Operational Modal Analysis Conference.

[37] Cole H. On-the-line analysis of random vibrations. Proceedings of the 9th Structural Dynamics And Materials Conference.

[38] Vesterholm K.K., Brincker R., Brandt A. (2020). Random decrement technique for detection and characterization of nonlinear behavior. Mech. Syst. Signal Process..

[39] Lin C., Chiang D.Y. (2012). A modified random decrement technique for modal identification from nonstationary ambi- ent response data only. J. Mech. Sci. Technol..

[40] Ku C.J., Cermak J.E., Chou L. (2007). Random decrement-based method for modal parameter identification of a dynamic system using acceleration responses. J. Wind Eng. Ind. Aerodyn..

[41] Torbol M. (2014). Real-Time Frequency-Domain Decomposition for Structural Health Monitoring Using General- Purpose Graphic Processing Unit. Comput.-Aided Civ. Infrastruct. Eng..

[42] Asmussen J.C. (1997). Modal Analysis Based on the Random Decrement Technique-Application to Civil Engineering Structures.

[43] Kasımzade A.A., Tuhta S., Aydin H., Günday F. Investigation of Modal Parameters on Steel Model Bridge Using EFDD Method. Proceedings of the 2nd International Conference on Technology and Science.

[44] Bedon C., Bergamo E., Izzi M., Noé S. (2018). Prototyping and Validation of MEMS Accelerometers for Structural Health Monitoring—The Case Study of the Pietratagliata Cable-Stayed Bridge. J. Sens. Actuator Netw..

[45] Komarizadehasl S., Lozano F., Lozano-Galant J.A., Ramos G., Turmo J. (2022). Low-Cost Wireless Structural Health Monitoring of Bridges. Sensors.

